# Identification and Analysis of Novel Amino-Acid Sequence Repeats in *Bacillus anthracis* str. *Ames* Proteome Using Computational Tools

**DOI:** 10.1155/2007/47161

**Published:** 2007-02-25

**Authors:** G. R. Hemalatha, D. Satyanarayana Rao, L. Guruprasad

**Affiliations:** School of Chemistry, University of Hyderabad, Hyderabad 500 046, India

## Abstract

We have identified four repeats and ten domains that are novel in proteins encoded by the *Bacillus anthracis* str. *Ames* proteome using automated in silico methods. A “repeat” corresponds to a region comprising less than 55-amino-acid residues that occur more than once in the protein sequence and sometimes present in tandem. A “domain” corresponds to a conserved region with greater than 55-amino-acid residues and may be present as single or multiple copies in the protein sequence. These correspond to (1) 57-amino-acid-residue PxV domain, (2) 122-amino-acid-residue FxF domain, (3) 111-amino-acid-residue YEFF domain, (4) 109-amino-acid-residue IMxxH domain, (5) 103-amino-acid-residue VxxT domain, (6) 84-amino-acid-residue ExW domain, (7) 104-amino-acid-residue NTGFIG domain, (8) 36-amino-acid-residue NxGK repeat, (9) 95-amino-acid-residue VYV domain, (10) 75-amino-acid-residue KEWE domain, (11) 59-amino-acid-residue AFL domain, (12) 53-amino-acid-residue RIDVK repeat, (13) (a) 41-amino-acid-residue AGQF repeat and (b) 42-amino-acid-residue GSAL repeat. A repeat or domain type is characterized by specific conserved sequence motifs. We discuss the presence of these repeats and domains in proteins from other genomes and their probable secondary structure.

## 1. INTRODUCTION

The anthrax is a disease of herbivores and other mammals including humans, caused by the *Bacillus anthracis* str. *Ames*, a Gram-positive, rod-shaped, nonmotile, spore-forming bacterium [1]. It is an endospore-forming bacterium that causes inhalational anthrax. During the course of disease, endospores are taken up by alveolar macrophages where they germinate in the phagolysosomal compartment. Vegetative cells then escape from the macrophage, eventually infecting blood. Expression of the major plasmid-encoded virulence determinants, tripartite toxin, and a poly-D-glutamic acid capsule is essential for full pathogenicity [2]. Key virulence genes found on plasmids are pXO1 and pXO2 [1]. The 60 MDa plasmid pXO2 carries genes required for the synthesis of an antiphagocytic poly-D-glutamic acid capsule [3]. The 110 MDa plasmid pXO1 [4] is required for the synthesis of the anthrax proteins, edema factor, lethal factor, and protective antigen. These proteins act in binary combinations to produce two anthrax toxins: edema toxin (a protective antigen and edema factor) and lethal toxin (a protective antigen and lethal factor) [5]. The chromosome encodes potential virulence factors that include haemolysins, enterotoxins, phospholipases, proteases, metalloproteases, and iron-acquisition proteins.

The chromosome of *B. anthracis* str. *Ames* contains three homologues of sortase transpeptidase that is responsible for attachment of secreted proteins to peptidoglycan on the cell surface of Gram-positive bacteria [6]. A range of important surface proteins, including enzymes and virulence-related MSCRAMMs (microbial surface components recognizing adhesive matrix molecules) are anchored to the cell wall in Gram-positive bacteria by sortase, a transpeptidase in *Staphylococcus aureus*, that cleaves polypeptides at a conserved LPxTG motif near the carboxyl terminus and covalently links them to penta-glycine crossbridges in peptidoglycan [7, 8]. Nearly 34 candidate surface proteins which have sortase attachment sites and SLH domains were identified. Two putative *B. anthracis* str. *Ames* sortase attached genes have internalin like repeats [9]. The chromosome of *B. anthracis* str. *Ames* also contains the *csaAB* genes for binding of proteins with S-layer homology (SLH) domains to polysaccharide. The SLH domain is a repetitive modular element that is present in several bacterial cell surface proteins and is involved in noncovalent association with peptidoglycan associated polymers [10]. The SLH domain comprises 55-amino-acid residues [11] and the potential role of most proteins with SLH domains on the surface of *B. anthracis* str. *Ames* is unknown at present [12]. However, these surface proteins may mediate unknown interactions between *B. anthracis* str. *Ames* and its external environment and could be targets for vaccine and drug design. Read et al. [12] reported the complete genome sequence of *B. anthracis* str. *Ames*. It comprises 5 227 293 base pairs and 5508 genes with an overall G+C content of 35.4%. Of these, 2762 are functional genes, 1212 are conserved hypothetical genes, 657 genes are of unknown function, and 877 genes are annotated as hypothetical proteins.

As the complete genome sequence of *B. anthracis* str. *Ames* is available [12], we intended to systematically identify and analyze all the amino-acid sequence repeats in this proteome. In a general context, a “repeat” corresponds to a region comprising less than 55-amino-acid residues that occur more than once, sometimes in tandem along the primary sequence, examples are the YVTN repeats in various cell surface proteins and the WD repeats present in proteins that perform a variety of functions. On the other hand, a “domain” refers to a region of the protein comprising greater than 55-amino-acid residues and does not contain internal sequence repeats. According to the crystallographer definition, a domain represents a region of the protein capable of folding independently as a stable unit. A domain can also exist in multiple copies and there can be several different domains per protein, examples are the SH2, SH3, and PH domains present in signal transduction proteins. The repeats and domains are characterized by conserved sequence motifs that may be identified according to the conservation of individual amino-acid residues at equivalent positions derived from multiple sequence alignments. In the absence of experimental data, the structural information can be obtained from secondary structure or fold prediction studies in silico. Information about the identified domains and repeats is represented in databases such as SMART, INTERPRO and PFAM. SMART (simple modular architecture research tool) allows the identification and annotation of genetically mobile domains and the analysis of domain architectures [13]. INTERPRO is a searchable database that provides information on sequence, function, and annotation. It is an integrated documentation resource for protein families, domains, and sites [14]. PFAM is a large collection of multiple sequence alignments and hidden Markov models covering many common protein domains and families. This can be used to view the domain organization of proteins [15]. We believe that a systematic sequence analysis will provide information on the novel repeats and domains present in *B. anthracis* str. *Ames* proteome that are not identified so far.

The *B. anthracis* str. *Ames* proteome consists of several known repeats and domains. Some of these domains are as follows. (1) BRCT (breast cancer carboxy terminal) domain was first identified as 100-amino-acid tandem repeat at the C-terminus of the tumor suppressor gene product BRCA1, in which the germline mutations lead to nearly 50% familial breast cancer. Most BRCT domains containing proteins participate in DNA damage checkpoint or DNA repair pathways and transcription regulation [16]. The BRCT is an evolutionarily conserved module that exists in a large number of proteins from prokaryotes to eukaryotes. (2) Excalibur (extracellular calcium binding) domain consists of a conserved DxDxDGxxCE motif, which is strikingly similar to the Ca^2+^ binding loop of the calmodulin like EF hand domains, suggesting an evolutionary relationship. (3) Cna_B domain forms a stalk in *Streptococcus aureus* collagen-binding protein that presents the ligand binding domain away from the bacterial cell surface. (4) CBS (cystathionine beta synthase) domain is a small intracellular module with 60-amino-acid residues, mostly found in two or four copies within a protein and occurs in several proteins in all kingdoms of life. Tandem pairs of CBS domains can act as binding domains for adenosine derivatives. In some cases, CBS domains may act as sensors of cellular energy status by being activated by AMP and inhibited by ATP. (5) Par B (par B like nuclease) domain cleaves single stranded DNA, nicks supercoiled plasmid DNA, and exhibits 5′-3′ exonuclease activity. (6) KH (K homology) domain comprises 70-amino-acids residues and is involved in RNA binding. (7) PAS and PAC domains comprising 300 and 45-amino-acid residues, respectively, mediate signal transduction. (8) PASTA domain is an extracellular module comprising 70-amino-acids residues that fold into a globular architecture consisting of 3*β*-strands and an *α*-helix which aids in penicillin binding. (9) NEAT (near transporter) domain is a 125-amino-acid residue conserved region consisting mainly *β*-strands. The NEAT domain appears to be associated with iron transport in several Gram-positive species, some of them are pathogenic. (10) SLH domain is present in several bacterial cell surface proteins and is involved in noncovalent association with peptidoglycan associated polymers. It comprises 55-amino-acid residues and the predicted secondary structure comprises two *α*-helices flanking a short *β*-strand [11].

The repeats present in *B. anthracis* str. *Ames* proteome are as follows. (1) RHS repeats are 21-amino-acids residues long and are involved in carbohydrate binding. (2) TPR (tetratricopeptide) repeats are 34-amino-acids residues long and are involved in protein-protein interactions. (3) EZ_−_HEAT repeats are 37–47-amino-acid residues long and occur in tandem in a number of cytoplasmic proteins that are involved in intracellular transport processes. Arrays of HEAT repeats consist of 3 to 36 units forming a rod-like helical structure and appear to function as protein-protein interaction surfaces. (4) Ankyrin repeats are about 33-amino-acid residues long and occur in at least four consecutive copies; the core of the repeat appears as a helix-loop-helix structure and is involved in protein-protein interactions. (5) LRR (lecuine rich repeats) are 20-amino-acids residues long, each repeat consists of a *β*-strand and *α*-helix, that are oriented in an antiparallel manner. The function of LRRs includes signal transduction, transmembrane receptors, DNA repair, cell adhesion, and extracellualr matrix proteins [17].

Andrade et al. [18] reviewed methods to identify repeats in proteins and the relationship between repeat sequences and their associated functions. Repeats may be identified by manual examination, if the sequence similarity is very high and present in tandem. Repeats are thought to arise due to gene duplication and recombination events. Protein domains may exist either as single or multiple copies and repeats always exist as multiple copies [18, 19]. Programs such as BLASTP [20] are also useful in detecting internal and homologous repeats in a protein database. By using the BLAST program, the presence of repeats in a query protein sequence can be identified if (a) the same region of the query is aligned against two or more distinct regions of a second protein; and (b) different regions of the query are being aligned against the same region of a second protein [18].

Several web-based methods are available for ab initio identification of sequence repeats in proteins. For example, RADAR (rapid automatic detection and alignment of repeats) [21] uses an automatic algorithm, for segmenting a query sequence into repeats; it identifies short composition biased as well as gapped approximate repeats and complex repeat architectures involving many different types of repeats in a query sequence. Rep program [22] uses an iterative algorithm based on score distributions from profile analysis. This procedure allows the identification of homologues at alignment scores lower than the highest optimal alignment score for nonhomologous sequences. The PROSPERO program [23] is ideal for large scale self-comparison of protein sequences. It uses a formula that accurately assesses the significance of protein repeat similarities, allowing for existence of gaps, and also takes into account sequence length and composition. TRUST (tracking repeats using significance and transitivity) program [24] exploits the concept of transitivity of alignments as well as a statistical scheme optimized for the evaluation of repeat significance. Starting from significant local suboptimal alignments, the application of transitivity allows to (1) identify distant repeat homologues for which no alignments were found; (2) gain confidence about consistently well-aligned regions; and (3) recognize and reduce the contribution of nonhomologous repeats. This assessment step will enable to derive a virtually noise-free profile representing a generalized repeat with high fidelity. It has been demonstrated by the authors that TRUST is a useful and reliable tool for mining tandem and nontandem repeats in protein sequence databases, to predict multiple repeat types with varying intervening segments within a single sequence. Once statistically significant repeats are detected, construction of a multiple sequence alignment provides insight into the extent of sequence homology among members of the new protein family and identification of the conserved sequence motifs.

We have implemented TRUST on a personal computer in our laboratory and used it to identify amino-acid sequence repeats in the proteins of *B. anthracis* str. *Ames* proteome. We have identified four repeats and ten domains that are novel in the proteome of *B. anthracis* str. *Ames*. Further analysis corresponding to searches of the completed and unfinished genome databases identified some of these to be present in other bacterial genomes.

## 2. METHODS

We have downloaded the entire proteome of *B. anthracis* str. *Ames* from the website http://www.ncbi.nlm.nih.gov in the FASTA format. The TRUST program was downloaded from the website and installed on the local Pentium IV computers on the Linux platform. The TRUST server together with the source code is available at http://ibivu.cs.vu.nl/programs/trustwww. The TRUST program was run for all the sequences in this proteome. Based on the size of the TRUST output file, the protein sequences with no internal repeats were discarded automatically; that is, only those protein sequences which comprise repeats were retained. The lengths of repeats and domains currently annotated in the INTERPRO database often comprise greater than 25-amino-acid residues; therefore, in this work, we have considered the repeats with greater than 25-amino-acid residues alone for further analysis. Thus selected proteins were submitted to SMART online (http://smart.embl-heidelberg.de/smart/batch.pl) [13] program in batch mode. Manual inspections of the SMART results identified proteins comprising known repeats or domains and were therefore discarded. Only those repeats that are not identified by SMART database are retained for further analysis.

**Figure 1:**
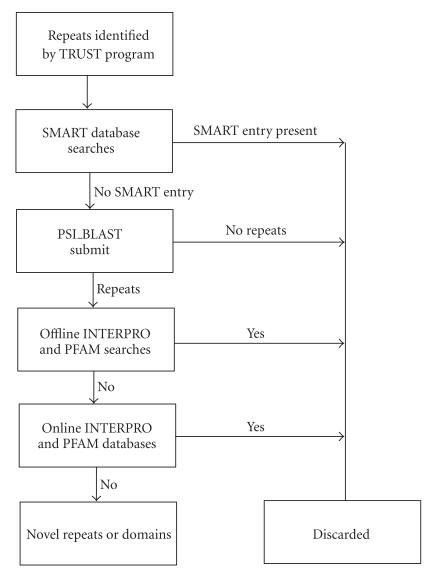
Flowchart for systematic analysis of repeats in proteins.

We have downloaded NCBI NR (release date: April 22, 2005) and UNIPROT (release date: April 23, 2005) databases and installed BLAST-2.2.10 on the local Linux computers (OS: Fedora Core-2, Pentium-IV 3.00 GHz, 1 GB RAM, 80 GB hard disk). Using automatic shell scripts, these protein sequences were then blasted using PSI-BLAST program [25] for three iterations against the NCBI NR database and using BLASTALL program against UNIPROT database. The proteins confirmed to comprise repeats by the BLAST program were retained and were tested for presence in the offline versions of INTERPRO (Database: iprscan_DATA_10.0, Applications: iprscan_V4.1, iprscan_binn4.x_Linux) and PFAM (release date: April 26, 2005) databases. A final check was made using online versions of INTERPRO and PFAM. These series of steps are given in the flowchart as shown in Figure 1.

The repeats which are not present in any of these databases were considered to be novel repeats or domains, depending upon (1) the number of times they occur in the protein sequences, and (2) length of the amino-acid sequence region. The novel repeats and domains thus identified in *B. anthracis* str. *Ames* proteome were subjected to PSI-BLAST analysis in order to identify other proteins from databases that comprise these repeats and domains. Multiple sequence alignment program, ClustalW [26], was used to detect the extent of sequence conservation and the secondary structure prediction was carried out using PHD [27] method.

## 3. RESULTS AND DISCUSSION

From the analysis of *B. anthracis* str. *Ames* proteome using TRUST program, we identified 905 proteins comprising of amino-acid sequence repeats. SMART database analysis identified that 302 entries do not have a SMART description. Based on their absence in the INTERPRO and PFAM databases and the length of repeat sequence (greater than 25-amino-acid residues), we have identified about 120 proteins (data not shown) in the *B. anthracis* str. *Ames* proteome to comprise novel amino-acid sequence repeats. We have added an additional constraint that the repeats identified by TRUST program should also be identified as a repeat by the BLAST program. Subsequent online INTERPRO and PFAM searches confirmed that these domains and repeats have not been reported before. In this work, we have identified four repeats and ten domains, that are not within or part of previously reported repeats and our findings are therefore novel. Further analysis identified some of these in the proteins of other bacterial genomes. The conserved amino-acid residues observed from multiple sequence alignments using the CLUSTALW program were used to describe sequence motifs characteristic of these novel repeats and domains. Often, more than one sequence motif is associated with repeats or domains and the amino-acid sequence patterns characteristic of these repeats are represented according to the PROSITE description [28]. Ponting et al. [29], have earlier used a similar approach to identify novel domains and repeats in *Drosophila melanogaster*.

In this work, we identified four repeats and ten domains that have not been reported before in the *B. anthracis* str. *Ames* proteome. The repeats and domains described in 1 to 6 and 9 are also present in some bacterial organisms, 7, 8, 10 and 11 are *Bacillus*-specific, 12 and 13 are *Bacillus anthracis* str. *Ames* specific. Lists of the proteins containing these novel repeats and domains are shown in Tables 1a to 1k. These tables indicate the protein identifiers (Gene or Swall_ID), the number of amino-acid residues in the protein, a description of the protein, and other well-characterized repeats and domains present in the protein. Some sequences representing these repeats or domains share lower than 15% pairwise sequence identity. However, these sequences retain the conserved motifs and the positions of secondary structure elements in the multiple sequence alignment. For all the proteins, the amino-acid sequence corresponding to each representative repeat are shown in the multiple sequence alignments (see Figures from 2 to 14).^1^ Conservation of the position of secondary structural elements is indicated from the multiple sequence alignment. The schematic figures used to represent these repeats and domains are shown in Figures 15 to 27. These figures (drawn to an approximate scale) reflect the relative proximity and location of individual repeats and domains along the primary sequence. We discuss each of these novel repeats and domains below.

### 3.1. 57-amino-acid-residue PxV domain

**Figure 2:**
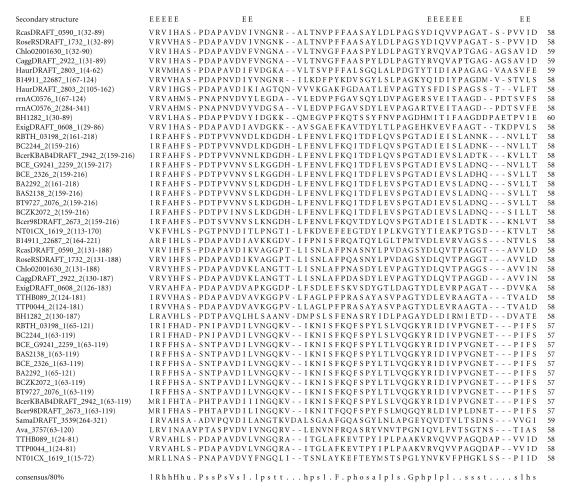
BA2292 is homologous to proteins GBAA0881 from *Bacillus anthracis* str. “*Ames* Ancestor.” BAS2138 is homologous to proteins BT9727_2076 from *Bacillus thuringiensis* serovar konkukian str. 97-27 and Bant_01002917 from *Bacillus anthracis* str. A2012.

The 251-amino-acid-residue protein corresponding to the GENE_ID BA2292 and described as hypothetical protein comprises of a 57-amino-acid-residue region as two copies. Further BLAST searches using sequence corresponding to the region (65–121) as a query identified 24 proteins that are described as hypothetical (see Table 1(a)). This region occurs as four copies in proteins from *Shewanella amazonensis, and Haloarcula marismortui*, as two copies in proteins from *B. anthracis, B. cereus, B. halodurans, B. thuringiensis, B. thuringiensis serovar, Thermus thermopilus, Chloroflexus aurantiacus, Chloroflexus aggregans Exiguobacterium sp., Bacillus weihenstephanensis, Roseiflexus castenholzii, Clostridium novyi, Herpetosiphon aurantiacus*, and as single copy in *Anabaena variabilis*; we therefore describe this region as a domain. The length of proteins varied between 196 to 488-amino-acid residues. The multiple sequence alignment corresponding to this domain is associated with PxV sequence motif where x is any amino-acid residue and is shown in Figure 2. The pairwise identities between sequences corresponding to PxV domain varied between 15–96%. The secondary structure corresponding to PxV domain is predicted to comprise four *β*-strands as shown in Figure 2. The representative domain architecture corresponding to proteins comprising the PxV domain is shown in Figure 15.

### 3.2. 122-amino-acid-residue FxF domain

**Figure 3:**
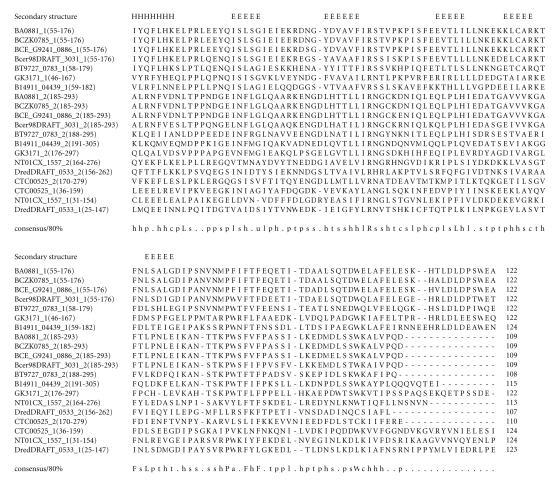
BA0881 is homologous to proteins GBAA0881 *Bacillus anthracis* str. “*Ames* Ancestor,” BAS0837 from *Bacillus anthracis* str. Sterne and Bant_01001534 from *Bacillus anthracis* str. A2012.

The 293-amino-acid-residue protein corresponding to the GENE_ID BA0881 and described as conserved domain protein comprises a 122-amino-acid-residue region as two copies. Further BLAST searches using sequence corresponding to the region (55–176) as a query identified 10 proteins (see Table 1(b)). The proteins comprising this region are described as either conserved or hypothetical proteins. This region occurs as two copies in the proteins of *B. anthracis, B. cereus, B. thuringiensis*, *Geobacillus kaustophilus, Clostridium tetani*, *Clostridium novyi*, and *Desulfotomaculum reducens* genomes. The length of proteins varied between 262 to 305-amino-acid residues. The multiple sequence alignment corresponding to this domain is associated with characteristic sequence motif FxF (Figure 3) and we refer to this as the FxF domain. The pairwise sequence identities corresponding to this domain varies between 18–97%. The secondary structure corresponding to FxF domain is predicted to comprise one *α*-helix and five *β*-strands, and the representative domain architecture of proteins comprising this domain is shown in Figure 16.

### 3.3. 111-amino-acid-residue YEFF domain

**Figure 4:**
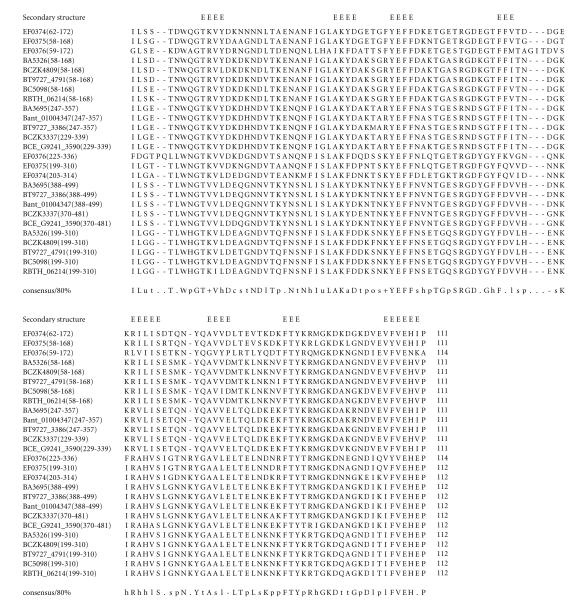
BA3695 is homologous to proteins GBAA3695 from *Bacillus anthracis* str. “*Ames* Ancestor” and BAS342 from *Bacillus anthracis* str. *Sterne*. BA5326 is homologous to proteins GBAA5326 from *Bacillus anthracis* str. “*Ames* Ancestor,” BAS4948 from *Bacillus anthracis* str. Sterne and Bant_01000199 from *Bacillus anthracis* str. A2012.

The 510-amino-acid-residue protein corresponding to the GENE_ID BA3695 and described as a S-layer protein comprises a 111-amino-acid-residue region that is present as two copies. Further BLAST searches, using sequence corresponding to the region (247–357) as a query, identified 13 proteins (see Table 1(c)), that are described as S-layer proteins, hypothetical, or lipoproteins and correspond to the *B. anthracis* str. *Ames* and A2012, *B. cereus*, *B. thuringiensis, B. thuringiensis serovar israelensis*, and *Enterococcus faecalis* genomes. The length of proteins varied between 321 to 510-amino-acid residues. Five proteins corresponding to the GENE_ID BA3695 and Bant_01004347 of *B. anthracis*, BCE_G9241_3590, and BCZK3337 of *B. cereus* and BT9727_3386 of *B. thuringiensis* comprise three copies of SLH domain, indicating a cell surface role for these proteins. This domain is characterized by conserved sequence motifs; YEFF, RGD, FTY, GKD, and FVEH. We refer to this 111-amino-acid region as the YEFF domain. The pairwise sequence identities corresponding to the YEFF domain varied between 36–96%. The consensus secondary structure predicted for this domain suggests mainly *β*-strands and the conserved sequence motifs, that is, YEFF and FTY are associated with *β*-strands; see Figure 4. The representative domain architecture of proteins comprising this domain is shown in Figure 17. It is intriguing that each domain comprises RGD sequence motif which is found in the proteins of extracellular matrix. Many viruses enter their host cells via the RGD motif—integrin interaction and synthetic peptides containing this RGD motif are active modulators of cell adhesion [30] . The RGD motif was originally identified as the sequence within fibronectin that mediates cell attachment. This motif has now been found in numerous other proteins and supports cell adhesion. The integrins, a family of cell surface proteins, act as receptors for cell adhesion molecules. A subset of the integrins recognizes the RGD motif within their ligands, the binding of which mediates both cell substratum and cell-cell interactions [31]. The presence of RGD motif and SLH domain implies that the YEFF domain comprising proteins is also present on the cell surface and mediates protein-protein interactions.

### 3.4. 109-amino-acid-residue IMxxH domain

**Figure 5:**
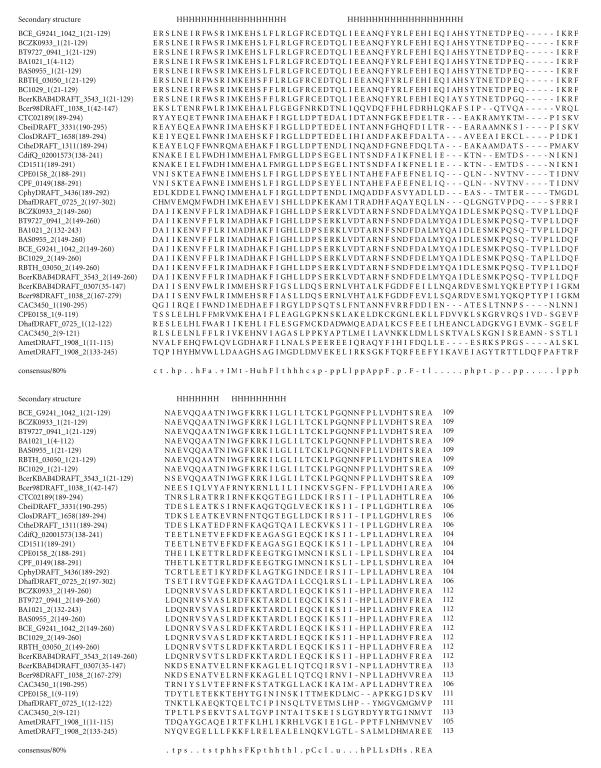
BAS0955 is homologous to proteins BT9727_0941 from *Bacillus thuringiensis* serovar konkukian str. 97-27, BCZK0933 from *Bacillus cereus* E33L, and BCE_G9241_1042 from *Bacillus cereus* G9241. BA1021 is homologous to protein GBAA1021 from *Bacillus anthracis* str. “*Ames* Ancestor.” BA0807 is homologous to proteins GBAA0807 from *Bacillus anthracis* str. “*Ames* Ancestor” and BAS0770 from *Bacillus anthracis* str. Sterne.

The 266-amino-acid-residue protein corresponding to the GENE_ID BA1021 and described as hypothetical protein comprises a 109-amino-acid-residue region as two copies. Further BLAST searches using sequence corresponding to the region (4–112) as a query identified 22 proteins (see Table 1(d)) that are described as either conserved or hypothetical proteins. This domain region occurs as two copies in all the proteins of *B. anthracis, B. cereus, B. thuringiensis, Bacillus weihenstephanensis C. acetobutylicum, C. perfringens, C. tetani, C. thermocellum, Desulfitobacterium hafniense, Clostridium phytofermentans,* and *Alkaliphilus metalliredigenes,* and as single domain in the 171-amino-acid-residue protein BcerKBAB4DRAFT_0307. The length of proteins varied between 171 to 321 amino acid residues. The multiple sequence alignment corresponding to this domain identified the characteristic sequence motifs; IMxxH, REA, and we refer to this as the IMxxH domain. The IMxxH sequence motif occurs at the N-terminal region of the domain. The pairwise sequence identities corresponding to the IMxxH domain varies between 5–98%. The secondary structure corresponding to IMxxH domain is predicted to comprise four *α*-helices as shown in Figure 5. The representative domain architecture corresponding to proteins comprising this domain is shown in Figure 18.

### 3.5. 103-amino-acid-residue VxxT domain

**Figure 6:**
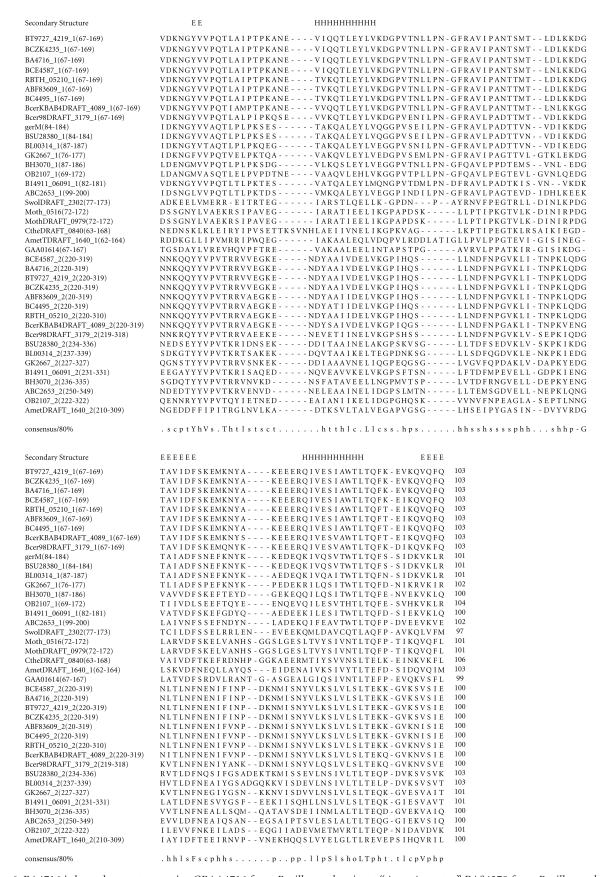
BA4716 is homologous to proteins GBAA4716 from *Bacillus anthracis* str. “*Ames* Ancestor,” BAS4378 from *Bacillus anthracis* str. Sterne, and Bant_01005366 from *Bacillus anthracis* str. A2012. BT9727_4219 is homologous to protein BCZK4235 from *Bacillus cereus* E33L. BA4716 is homologous to protein BL02986 from *Bacillus licheniformis* ATCC 14580.

The 349-amino-acid-residue protein corresponding to the GENE_ID BA4716 and described as germination protein comprises a 103-amino-acid-residue region as two copies. Further BLAST searches using sequence corresponding to the region (67–169) as query identified 23 proteins (see Table 1(e)). The proteins comprising this domain are described as germination proteins as the *Bacillus anthracis* is an endospore-forming bacterium. This domain region occurs twice in proteins of *B. anthracis* str. *Ames, B. cereus, B. clausii, B. thuringiensis*, *B. thuringiensis serovar israelensis*, *Alkaliphilus metalliredigene*, and *Bacillus weihenstephanensis* genomes and only once in the proteins of *Syntrophomonas wolfei str. Goettingen, Moorella thermoacetica, Clostridium thermocellum, B. subtilis*, and *Pelotomaculum thermopropionicum* genomes. The length of proteins varied between 195 to 377-amino-acid residues. The multiple sequence alignment corresponding to this domain identified VxxT as sequence motif. This sequence motif occurs in the N-terminal region of each protein and the pairwise sequence identity varied between 11–98%. The secondary structure is predicted to comprise two *α*-helices and three *β*-strands as shown in Figure 6. The representative domain architecture corresponding to proteins comprising this domain is shown in Figure 19.

### 3.6. 84-amino-acid-residue ExW domain

**Figure 7:**
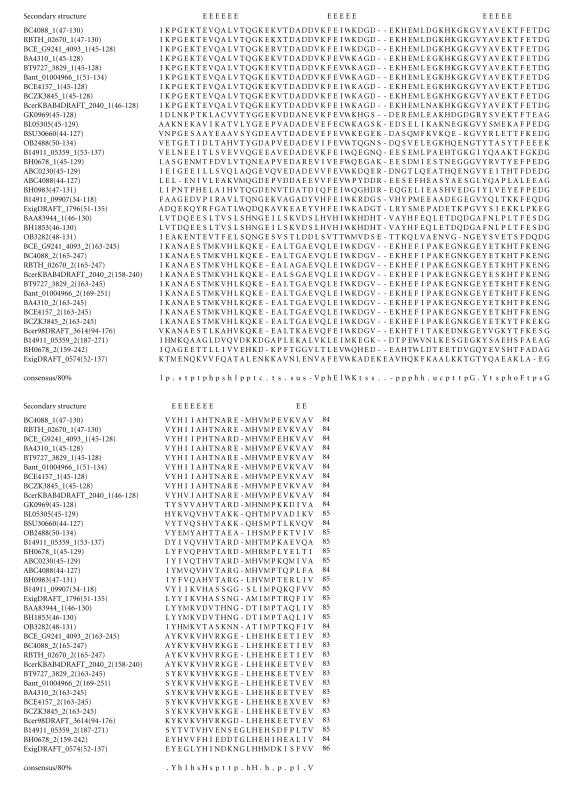
BA4310 is homologous to proteins GBAA4310 from *Bacillus anthracis* str. “*Ames* Ancestor,” BAS3998 from *Bacillus anthracis* str. Sterne, and BT9727_3829 from *Bacillus thuringiensis* serovar konkukian str. 97-27.

The 246-amino-acid-residue protein corresponding to the GENE_ID BA4310 and described as hypothetical protein comprises an 84-amino-acid-residue region as two copies. Further BLAST searches using sequence corresponding to the domain (45–128) as a query identified 25 proteins (Table 1(f)) that are described as either conserved or hypothetical proteins. This domain region occurs as two copies in proteins of *B. anthracis* str. *Ames, B. cereus, B. halodurans* (GENE_ID BH0678), *B. thuringiensis*, *B. thuringiensis serovar israelensis*, *Geobacillus kaustophil*us, *Bacillus weihenstephanensis*, and *Exiguobacterium sibiricum* genomes and as single copy in proteins of *B. clausii*, *B. halodurans* (GENE_ID BH0983), *B. licheniformis, B. subtilis, Exiguobacterium sp.*, and *Oceanobacillus ihenyensis* genomes. The length of proteins varied between 142 to 273-amino-acid residues. The multiple sequence alignment corresponding to this domain identified ExW sequence motif. The pairwise sequence identities corresponding to the ExW domain varied between 14–98%. The secondary structure of this domain is predicted to comprise five *β*-strands and the conserved sequence motif is associated with one of the *β*-strands as shown in Figure 7. The representative domain architecture corresponding to proteins comprising this domain is shown in Figure 20.

### 3.7. 104-amino-acid-residue NTGFIG domain

**Figure 8:**
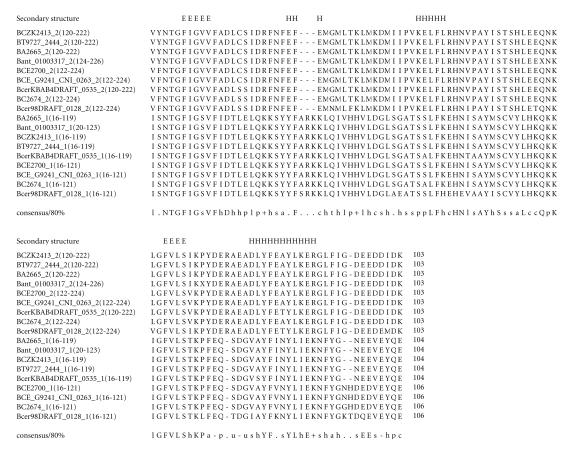
BA2665 is homologous to proteins GBAA2665 from *Bacillus anthracis* str. “*Ames* Ancestor,” BAS2482 from *Bacillus anthracis* str. Sterne. BT9727_2444 is homologous to protein BCZK2413 from *Bacillus cereus* E33L.

The 232-amino-acid-residue protein corresponding to the GENE_ID BA2665 and described as a hypothetical protein comprises a 104-amino-acid-residue region as two copies in tandem. Further BLAST searches using sequence corresponding to the region (16–119) as query identified 9 hypothetical proteins comprising this domain from organisms such as *B. anthracis*, *B. thuringiensis, Bacillus weihenstephanensis*, and *B. cereus*. The protein corresponding to the GENE_ID BCZK2413 of *B. cereus* is described as group-specific protein. The list of 9 proteins comprising this domain is shown in Table 1(g). The length of proteins varied between 232 to 236-amino-acid residues. This domain occurs twice in every protein of the bacillus species as shown in Table 1(g). We refer to this as the NTGFIG domain based on the conserved sequence motif that is present at the N-terminal part. The pairwise sequence identities between sequences corresponding to this domain varied between 31–99%. The secondary structure corresponding to this domain is predicted to comprise three *α*-helices and two *β*-strands as shown in Figure 8. The representative domain architecture corresponding to proteins comprising this domain is shown in Figure 21.

### 3.8. 36-amino-acid-residue NxGK repeat

**Figure 9:**
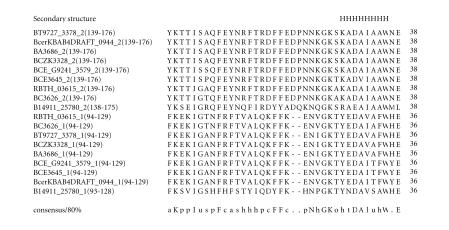
BT9727_3378 is homologous to protein BCZK3328 from *Bacillus cereus* E33L. BA3686 is homologous to proteins GBAA3686 from *Bacillus anthracis* str. “*Ames* Ancestor,” BAS3417 from *Bacillus anthracis* str. Sterne, and Bant_01004341 from *Bacillus anthracis* str. A2012.

The 193-amino-acid-residue protein corresponding to GENE_ID BA3686 and described as hypothetical cytosolic protein comprises a 36-amino-acid-residue region as two copies. Further BLAST searches using sequence corresponding to the region (94–129) as query identified 9 hypothetical proteins comprising this repeat region from the organisms *B. anthracis*, *B. thuringiensis*, *B. thuringiensis serovar israelensis, Bacillus weihenstephanensis*, and *B. cereus* (see Table 1(h)). The length of proteins varied between 189 to 193-amino-acid residues, and also consists a SAP domain at the N-terminus, in addition to the novel repeat described here. A SAP domain consists of two *α*-helices and is a DNA-binding motif that is involved in chromosomal organization [32]. Therefore, we believe that these repeats might also participate in a similar function. The multiple sequence alignment corresponding to this repeat identified NxGK sequence motif (Figure 9). The pairwise sequence identities between sequences corresponding to NxGK repeats varied between 36–97%. The secondary structure is predicted to comprise a *α*-helix and the conserved sequence motif described above is also associated with *α*-helix. The representative domain architecture corresponding to proteins comprising the NxGK repeats is shown in Figure 22.

### 3.9. 95-amino-acid-residue VYV domain

**Figure 10:**
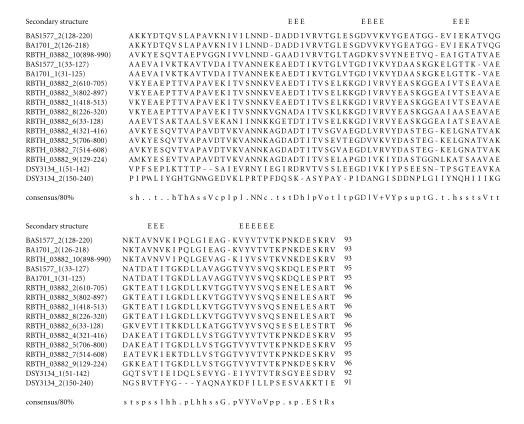
BA1701 is homologous to proteins GBAA1701 from *Bacillus anthracis* str. “*Ames* Ancestor,” and Bant_01002313 from *Bacillus anthracis* str. A2012.

The 225-amino-acid-residue protein corresponding to the GENE_ID BA1701 and described as a hypothetical protein comprises a 95-amino-acid-residue region, as two copies in tandem. Further BLAST searches using sequence corresponding to the region (31–125) as query identified BAS1577 protein of *B. anthracis*, RBTH_03882 protein of *Bacillus thuringiensis serovar* *israelensis*, and DSY3134 of *Desulfitobacterium hafniense* Y51 that are described as hypothetical proteins. The length of proteins varied between 227 to 1674-amino-acid residues (see Table 1(i)). In RBTH_03882, this region occurs ten times and in tandem. The multiple sequence alignment corresponding to this domain identified characteristic sequence motifs; GDxV, VYV (see Figure 10). For the sake of simplicity, we refer to this 95-amino-acid region as VYV domain. The pairwise sequence identities between sequences corresponding to VYV domains varied between 29–95%. The secondary structure corresponding to VYV domain is predicted to comprise five *β*-strands. The representative domain architecture corresponding to proteins comprising the VYV domains is shown in Figure 23.

### 3.10. 75-amino-acid-residue KEWE domain

**Figure 11:**
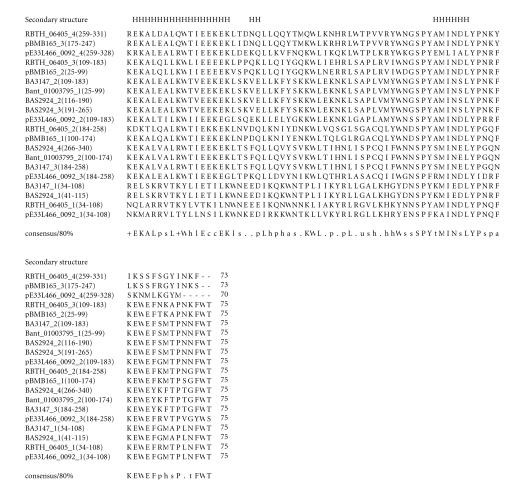
BA3147 is homologous to protein GBAA3147 from *Bacillus anthracis* str. “*Ames* Ancestor”.

The 262-amino-acid-residue protein corresponding to the GENE_ID BA3147 and described as a hypothetical protein comprises a 75-amino-acid-residue region as three copies in tandem. Further BLAST searches using the sequence corresponding to the region (34–108) as query identified this domain in 6 proteins that are described as hypothetical proteins (see Table 1(j)). This domain may exist as 2, 3, or 4 copies in these proteins. The length of proteins identified varied between 178 to 344-amino-acid residues. The pairwise sequence identities between sequences corresponding to these regions varied between 22–69%. These domains are present in tandem and associated with SPY, MIN, LYP, KEWE, and FWT conserved sequence motifs as indicated in the multiple sequence alignment (see Figure 11). We refer to these as the KEWE domain, and this sequence motif occurs at the C-terminus of the domain. The secondary structure corresponding to KEWE domain is predicted to comprise three *α*-helices as shown in Figure 11. The representative domain architecture corresponding to proteins comprising the KEWE domain is shown in Figure 24.

### 3.11. 59-amino-acid-residue AFL domain

**Figure 12:**
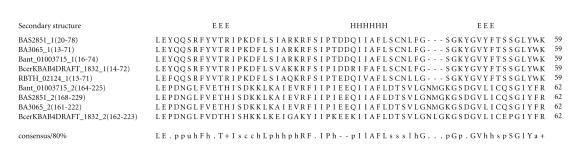
BA3065 is homologous to protein GBAA3065 from *Bacillus anthracis* str. “*Ames* Ancestor”.

The 290-amino-acid-residue protein corresponding to the GENE_ID BA3065 and described as hypothetical protein comprises a 59-amino-acid-residue region as two copies. Further BLAST searches using sequence corresponding to the region (13–71) as query identified that this region occurs twice in the proteins with GENE_ID’s: BAS2851 and Bant_01003715 of *B. anthracis* strains, the protein with GENE_ID: BcerKBAB4DRAFT_1832 of *Bacillus weihenstephanensis,* and once in the protein with GENE_ID: RBTH_02124 of *Bacillus thuringiensis serovar israelensis* (see Table 1(K)). The lengths of the proteins varied between 145 to 297-amino-acid residues and are described as hypothetical proteins. The multiple sequence alignment corresponding to this domain identified two characteristic sequence motifs: RFxI and AFL (see Figure 12). We refer to this as the AFL domain. The sequence identities shared between AFL domains varied between 38–91%. The secondary structure corresponding to the AFL domain is predicted to comprise of one *α*-helix and two *β*-strands and the conserved sequence motif AFL is a part of the *α*-helix. The representative domain architecture corresponding to protein comprising the AFL domain is shown in Figure 25.

### 3.12. 53-amino-acid-residue RIDVK repeat

**Figure 13:**
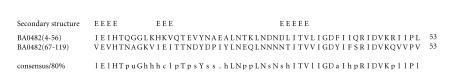
BA0482 is homologous to proteins GBAA0482 from *Bacillus anthracis* str. “*Ames* Ancestor,” BAS0458 from *Bacillus anthracis* str. Sterne, and Bant_01001108 from *Bacillus anthracis* str. A2012.

The 159-amino-acid-residue protein corresponding to the GENE_ID BA0482 and described as a conserved domain protein comprises a 53-amino-acid region as two copies. BLAST did not identify this repeat in any other proteins; therefore this repeat is unique to *B. anthracis* str. *Ames*. The multiple sequence alignment corresponding to this repeat identified three characteristic sequence motifs: ITV, IGD, and RIDVK (Figure 13). We refer to this as the RIDVK repeat. The sequence identity shared between this RIDVK repeats in BA0482 is 45%. The secondary structure corresponding to the RIDVK repeat is predicted to comprise three *β*-strands. The representative domain architecture corresponding to protein comprising the RIDVK repeat is shown in Figure 26.

### 3.13. (a) 41-amino-acid-residue AGQF repeat and (b) 42-amino-acid-residue GSAL repeat

The protein corresponding to the GENE_ID BA4081 comprises 462 amino acid residues and described as conserved domain protein contains two novel repeat types. The sequence length corresponding to repeat types are 41 and 42 amino acid residues and are present as two copies in BA4081. BLAST searches identified these repeats to be specific to this protein alone.

**Figure 14:**
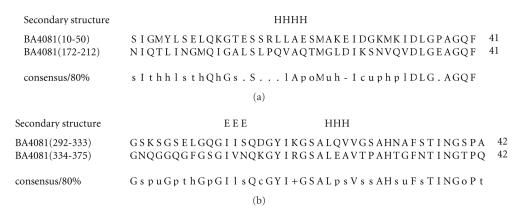
BA4081 is homologous to proteins GBAA4081 from *Bacillus anthracis* str. “*Ames* Ancestor,” BAS3792 from *Bacillus anthracis* str. Sterne, and Bant_01004731 from *Bacillus anthracis* str. A2012.

**Figure 15:**
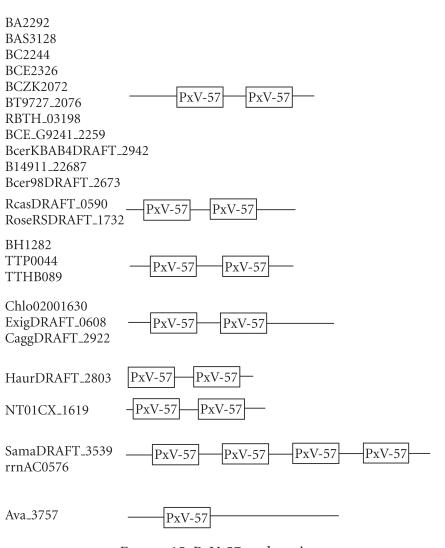
PxV-57 aa domain.

**Figure 16:**
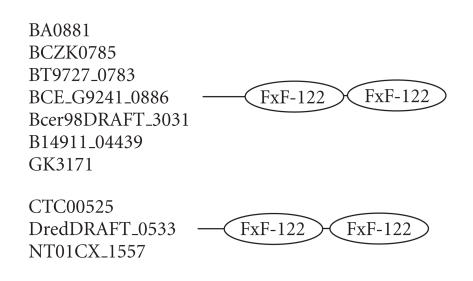
FXF-122 aa domain.

**Figure 17:**
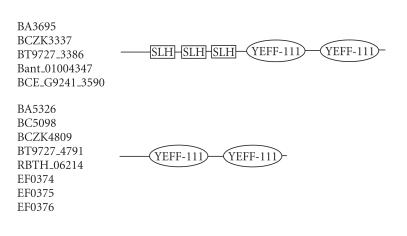
YEFF-111 aa domain.

**Figure 18:**
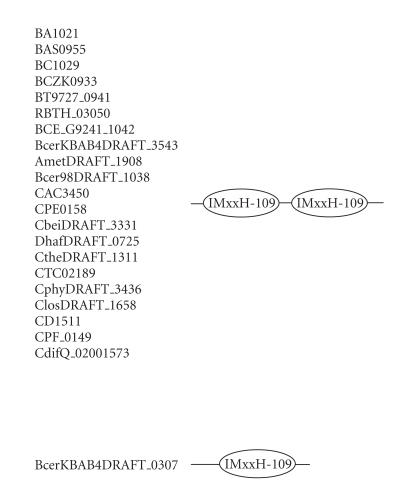
IMxxH-109 aa domain.

**Figure 19:**
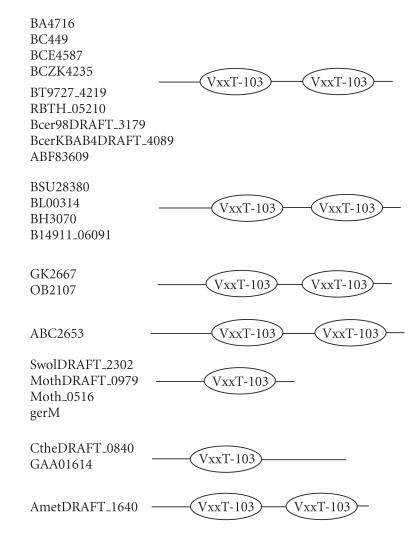
VxxT-103 aa domain.

**Figure 20:**
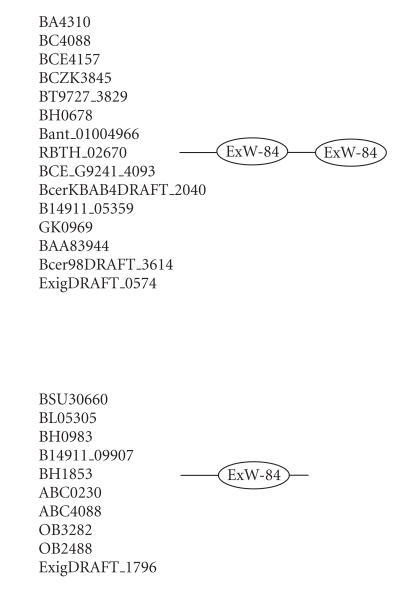
ExW-84 aa domain.

**Figure 21:**
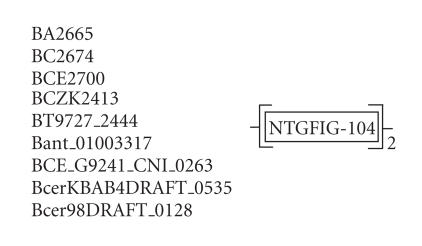
NTGFIG-104 aa domain.

**Figure 22:**
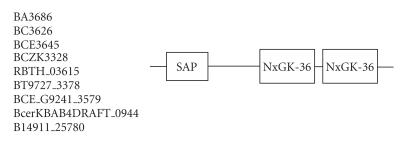
NxGK-36 aa repeat.

**Figure 23:**
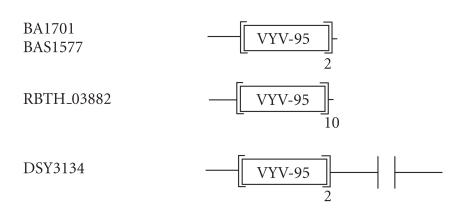
VYV-95 aa domain.

**Figure 24:**
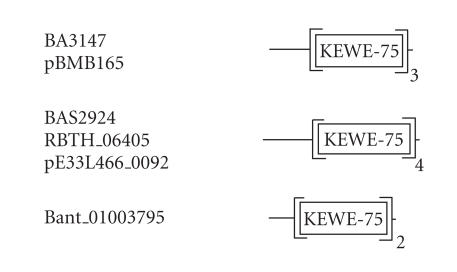
KEWE-75 aa domain.

**Figure 25:**
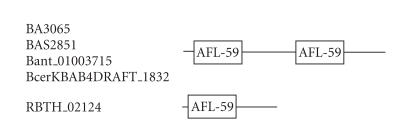
AFL-59 aa domain.

**Figure 26:**
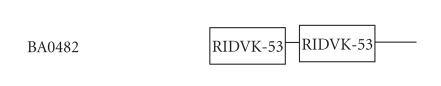
RIDVK-53 aa repeat.

**Figure 27:**
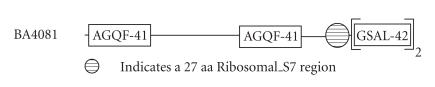
AGQF-41 aa repeat; GSAL-42 aa repeat.

(a) The sequence alignment corresponding to 41-amino-acid-residue repeat identified two characteristic sequence motifs: DLG and AGQF (Figure 14(a)). We refer to this as the AGQF repeat. The motif occurs at the C-terminal part of the repeat region. The sequence homology shared between this AGQF repeats is about 34%. The secondary structure corresponding to the AGQF repeat is predicted to comprise one *α*-helix. The representative domain architecture corresponding to protein comprising the AGQF repeat is shown in Figure 27.

(b) The sequence alignment corresponding to the 42-amino-acid-residue tandem repeat identified three characteristic sequence motifs: GYI, GSAL, and TING (Figure 14(b)) and is a glycine-rich repeat. We refer to this as the GSAL repeat. The sequence homology shared between this GSAL repeats is 52%. The secondary structure corresponding to the GSAL repeat is predicted to comprise one *α*-helix and one *β*-strand. The representative domain architecture corresponding to protein comprising the GSAL repeat is shown in Figure 27. This protein is associated with a 27-amino-acid residue Ribosomal_S7 region that is sandwiched between the 41-amino-acid-residue AGQF repeat and the 42-amino-acid-residue GSAL repeat. These two repeats are specific to this protein alone and are therefore *B. anthracis* str. *Ames* specific.

From the analysis of the *B. anthracis* proteome, we observed that the novel repeats and domains are present in all the strains, such as *Ames*, *Ames* ancestor, Sterne, and A2012, that have been sequenced so far. This indicates that these strains of *B. anthracis* have diverged recently. We also observed that the domains PxV, FxF, YEFF, VxxT, ExW, and VYV are present in proteins from several bacterial organisms. The domains NTGFIG, KEWE, AFL, and the repeats NxGK are specific to bacillus. It is interesting to note that the domains VYV and AFL are present in all the *B. anthracis* species while absent in *B. cereus* genomes. The repeats RIDVK, AGQF, and GSAL are also specifically present only in all the strains of *B. anthracis*. This analysis explains some differences in the closely related *B. anthracis* and *B. cereus* genomes. The identification of these novel domains and repeats in subsequently sequenced genomes will add value to their annotation.

## 4. CONCLUSIONS

A systematic analysis using computational tools identified four novel repeats and ten domains corresponding to the *B. anthracis* str. *Ames* proteome. Further database searches identified that some novel repeats and domains are also present in other bacterial genomes. The NxGK repeats are associated with SAP domain. The SAP domain is a DNA-binding motif that is involved in chromosomal organization. Therefore, we believe that these repeats also participate in similar function. The YEFF domain containing proteins are associated with RGD motif and may be involved in cell adhesion. The identification of novel repeats and domains corresponding to *B. anthracis* proteome may be useful for annotation. From the presence of VYV and AFL domains in all the *B. anthracis* species and their absence in *B. cereus* genomes, we identified some differences in these two genomes that are otherwise closely related.

## Figures and Tables

**Table:** (a) List of proteins containing the 57-amino-acid-residue PxV domain.

Gene ID (number of residues)	Organism	Description	Number of PxV domains

BA2292 (251)	*Bacillus anthracis* str. *Ames* (B)	Hypothetical protein	2
BAS2138 (249)	*Bacillus anthracis* Sterne (B)	Hypothetical protein	2
BT9727_2076 (249)	*Bacillus thuringiensis* serovar konkukian str. 97-27 (B)	Hypothetical protein	2
BCZK2072 (249)	*Bacillus cereus* E33L (B)	Hypothetical protein	2
BCE2326 (249)	*Bacillus cereus* ATCC 10987 (B)	Hypothetical protein	2
BC2244 (249)	*Bacillus cereus* ATCC 14579 (B)	Hypothetical protein	2
BH1282 (222)	*Bacillus halodurans* C-125 (B)	BH1282 protein	2
BCE_G9241_2259 (249)	*Bacillus cereus* G9241 (B)	Hypothetical conserved protein	2
RBTH_03198 (251)	*Bacillus thuringiensis* serovar israelensis ATCC 35646 (B)	Hypothetical protein	2
TT_P0044 (221)	*Thermus thermopilus* HB27 (B)	Hypothetical conserved protein	2
TTHB089 (221)	*Thermus thermophilus* HB8 (B)	Hypothetical protein	2
Chlo02001630 (262)	*Chloroflexus aurantiacus* J-10-fl (B)	Hypothetical protein	2
ExigDRAFT__0608 (264)	*Exiguobacterium* sibiricum 255-15 (B)	Hypothetical protein	2
SamaDRAFT_3539 (469)	*Shewanella amazonensis* SB2B (B)	Hypothetical protein	4
rrnAC0576 (488)	*Haloarcula marismortui* ATCC 43049 (A)	Unknown	4
Ava_3757 (292)	*Anabaena variabilis* ATCC 29413 (B)	Hypothetical protein	1
BcerKBAB4DRAFT_2942 (249)	*Bacillus weihenstephanensis* KBAB4 (B)	Conserved hypothetical protein	2
B14911_22687 (254)	*Bacillus* sp. NRRL B-14911 (B)	Hypothetical protein	2
Bcer98DRAFT_2673 (249)	*Bacillus cereus* subsp. cytotoxis NVH (B)	Conserved hypothetical protein	2
RcasDRAFT_0590 (259)	*Roseiflexus castenholzii* DSM 13941 (B)	Surface protein from Gram-positive cocci, anchor region	2
RoseRSDRAFT_1732 (259)	*Roseiflexus* sp. RS-1 (B)	Surface protein from Gram-positive cocci, anchor region	2
NT01CX_1619 (210)	*Clostridium novyi* NT (B)	Conserved hypothetical protein	2
HaurDRAFT_2803 (196)	*Herpetosiphon aurantiacus* ATCC 23779 (B)	Conserved hypothetical protein	2
CaggDRAFT_2922 (261)	*Chloroflexus aggregans* DSM 9485 (B)	Conserved hypothetical protein	2

**Table:** (b) List of proteins containing the 122-amino-acid-residue FxF domain.

Gene ID (number of residues)	Organism	Description	Number of FxF domains

BA0881 (293)	*Bacillus anthracis* str. *Ames* (B)	Conserved domain protein	2
BCZK0785 (293)	*Bacillus cereus* E33L (B)	Hypothetical protein	2
BT9727_0783 (295)	*Bacillus thuringiensis* serovar konkukian str. 97-27 (B)	Hypothetical protein	2
BCE_G9241_0886 (293)	*Bacillus cereus* G9241 (B)	Conserved protein, putative	2
GK3171 (297)	*Geobacillus kaustophilus* HTA426 (B)	Hypothetical conserved protein	2
CTC00525 (279)	*Clostridium tetani* E88 (B)	Hypothetical protein	2
Bcer98DRAFT_3031 (293)	*Bacillus cereus* subsp. cytotoxis NVH (B)	Conserved hypothetical protein	2
B14911_04439 (305)	*Bacillus* sp. NRRL B-14911 (B)	Hypothetical protein	2
DredDRAFT_0533 (262)	*Desulfotomaculum reducens* MI-1 (B)	Hypothetical protein	2
NT01CX_1557 (276)	*Clostridium novyi* NT (B)	Conserved protein, putative	2

**Table:** (c) List of proteins containing the 111-amino-acid-residue YEFF domain.

Gene ID (number of residues)	Organism	Description and other known domains	Number of YEFF domains

BA3695 (510)	*Bacillus anthracis* str. *Ames* (B)	S-layer protein, putative, SLH-domain (3)	2
BCZK3337 (492)	*Bacillus cereus* E33L (B)	S-layer protein, SLH-domain (3)	2
BT9727_3386 (510)	*Bacillus thuringiensis* serovar konkukian str. 97-27 (B)	S-layer protein, SLH-domain (3)	2
Bant_01004347 (510)	*Bacillus anthracis* str. A2012 (B)	Hypothetical protein, SLH-domain (3)	2
BCE_G9241_3590 (492)	*Bacillus cereus* G9241 (B)	Lipoprotein, putative SLH-domain (3)	2
BA5326 (321)	*Bacillus anthracis* str. *Ames* (B)	Lipoprotein, putative	2
BT9727_4791 (321)	*Bacillus thuringiensis* serovar konkukian str. 97-27 (B)	Hypothetical protein	2
BC5098 (321)	*Bacillus cereus* ATCC 14579 (B)	Hypothetical protein	2
BCZK4809 (321)	*Bacillus cereus* E33L (B)	Hypothetical protein	2
RBTH_06214 (321)	*Bacillus thuringiensis* serovar israelensis ATCC 35646 (B)	Hypothetical protein	2
EF0374 (325)	*Enterococcus faecalis* V583 (B)	Lipoprotein, putative	2
EF0375 (321)	*Enterococcus faecalis* V583 (B)	Hypothetical protein	2
EF0376 (347)	*Enterococcus faecalis* V583 (B)	Hypothetical protein	2

**Table:** (d) List of proteins containing the 109-amino-acid-residue IMxxH domain.

Gene ID (number of residues)	Organism	Description	Number of IMxxH domains

BA1021 (266)	*Bacillus anthracis* str. *Ames* (B)	Hypothetical protein	2
BAS0955 (283)	*Bacillus anthracis* Sterne (B)	Hypothetical protein	2
BCZK0933 (283)	*Bacillus cereus* E33L (B)	Hypothetical protein	2
BT9727_0941 (283)	*Bacillus thuringiensis* serovar konkukian str. 97-27 (B)	Hypothetical protein	2
BC1029 (283)	*Bacillus cereus* ATCC 14579 (B)	Hypothetical protein	2
RBTH_03050 (283)	*Bacillus thuringiensis* serovar israelensis ATCC 35646 (B)	Hypothetical protein	2
CAC3450 (307)	*Clostridium acetobutylicum* ATCC 824 (B)	Hypothetical protein	2
CPE0158 (303)	*Clostridium perfringens* str. 13 (B)	Hypothetical protein	2
CTC02189 (314)	*Clostridium tetani* E88 (B)	Conserved protein	2
CtheDRAFT_1311 (307)	*Clostridium thermocellum* ATCC 27405 (B)	Conserved hypothetical protein	2
DhafDRAFT_0725 (321)	*Desulfitobacterium hafniense* DCB-2 (B)	Conserved hypothetical protein	2
BCE_G9241_1042 (283)	*Bacillus cereus* G9241 (B)	Conserved protein	2
CbeiDRAFT_3331 (312)	*Clostridium beijerincki* NCIMB 8052 (B)	Conserved hypothetical protein	2
CphyDRAFT_3436 (305)	*Clostridium phytofermentans* ISDg (B)	Conserved hypothetical protein	2
ClosDRAFT_1658 (308)	*Clostridium* sp. OhILAs (B)	Conserved hypothetical protein	2
CdifQ_02001573 (254)	*Clostridium difficile* QCD-32g58 (B)	Hypothetical protein	2
BcerKBAB4DRAFT_3543 (283)	*Bacillus weihenstephanensis* KBAB4 (B)	Hypothetical protein	2
AmetDRAFT_1908 (272)	*Alkaliphilus metalliredigenes* QYMF (B)	Conserved hypothetical protein	2
CD1511 (304)	*Clostridium difficile* 630 (B)	Conserved hypothetical protein	2
CPF_0149 (303)	*Clostridium perfringens* ATCC 13124 (B)	Hypothetical protein	2
BcerKBAB4DRAFT_0307 (171)	*Bacillus weihenstephanensis* KBAB4 (B)	Conserved hypothetical protein	1
Bcer98DRAFT_1038 (303)	*Bacillus cereus* subsp. cytotoxis NVH 391-98 (B)	Conserved hypothetical protein	2

**Table:** (e) List of proteins containing the 103-amino-acid-residue VxxT domain.

Gene ID (number of residues)	Organism	Description	Number of VxxT domains

BA4716 (349)	*Bacillus anthracis* str. *Ames* (B)	Germination protein gerM	2
gerM BT9727_4219 (349)	*Bacillus thuringiensis* serovar konkukianstr. 97-27 (B)	Germination protein	2
germ BCZK4235 (349)	*Bacillus cereus* E33L (B)	Germination protein	2
BCE4587 (349)	*Bacillus cereus* ATCC 10987 (B)	Germination protein gerM	2
BC4495 (349)	*Bacillus cereus* ATCC 14579 (B)	Germination protein germ	2
BSU28380 (366)	*Bacillus subtilis* subsp. subtilis str. 168 (B)	Germination protein gerM	2
BL00314 (369)	*Bacillus licheniformis* ATCC 14580 (B)	Spore germination protein GerM	2
BH3070 (365)	*Bacillus halodurans* C-125 (B)	Germination (Cortex hydrolysis) and sporulation	2
RBTH_05210 (349)	*Bacillus thuringiensis* serovar israelensis ATCC 35646 (B)	Germination protein germ	2
gerM (210)	*Bacillus subtilis* (B)	gerM	1
ABC2653 (377)	*Bacillus clausii* KSM-K16 (B)	Germination protein GerM	2
GK2667 (357)	*Geobacillus kaustophilus* HTA426 (B)	Germination (Cortex hydrolysis) and sporulation	2
OB2107 (352)	*Oceanobacillus iheyensis* HTE831 (B)	Germination (Cortex hydrolysis) and sporulation	2
SwolDRAFT_2302 (195)	*Syntrophomonas wolfei* str. Goettingen (B)	Hypothetical protein	1
MothDRAFT_0979 (200)	*Moorella thermoacetica* ATCC 39073 (B)	Similar to Spore germination protein	1
CtheDRAFT_0840 (299)	*Clostridium thermocellum* ATCC 27405 (B)	Hypothetical protein	1
gerM ABF83609 (349)	*Bacillus thuringiensis* serovar kurstaki (B)	Spore germination protein	2
Bcer98DRAFT_3179 (348)	*Bacillus cereus* subsp. cytotoxis NVH 391-98 (B)	Germination protein GerM	2
BcerKBAB4DRAFT_4089 (349)	*Bacillus weihenstephanensis* KBAB4 (B)	Germination protein gerM	2
B14911_06091 (361)	*Bacillus* sp. NRRL B-14911 (B)	Spore germination protein	2
GAA01614 (295)	*Pelotomaculum thermopropionicum* SI (B)	Unnamed protein product	1
AmetDRAFT_1640 (332)	*Alkaliphilus metalliredigenes* QYMF (B)	Hypothetical protein	2
Moth_0516 (200)	*Moorella thermoacetica* ATCC 39073 (B)	Spore germination protein-like	1

**Table:** (f) List of proteins containing the 84-amino-acid-residue ExW domain.

Gene ID (number of residues)	Organism	Description	Number of ExW domains

BA4310 (246)	*Bacillus anthracis* str. *Ames* (B)	Hypothetical protein	2
BT9727_3829 (246)	*Bacillus thuringiensis* serovar konkukian str. 97-27 (B)	Hypothetical protein	2
BCE4157 (246)	*Bacillus cereus* ATCC 10987 (B)	Hypothetical protein	2
BCZK3845 (246)	*Bacillus cereus* E33L (B)	Hypothetical protein	2
BC4088 (248)	*Bacillus cereus* ATCC 14579 (B)	IG hypothetical 17224	2
GK0969 (226)	*Geobacillus kaustophilus* HTA426 (B)	Hypothetical conserved protein	2
BSU30660 (145)	*Bacillus* subtilis subsp. str. 168 (B)	Hypothetical protein ytkA (PSPA8)	1
BL05305 (147)	*Bacillus licheniformis* ATCC 14580 (B)	Conserved protein YtkA	1
BH0983 (157)	*Bacillus halodurans* C-125 (B)	BH0983 protein	1
Bant_01004966 (252)	*Bacillus anthracis* str. A2012 (B)	Protein chain release factor A	2
RBTH_02670 (248)	*Bacillus thuringiensis* serovar israelensis ATCC 35646 (B)	Hypothetical protein	2
BCE_G9241_4093 (246)	*Bacillus cereus* G9241 (B)	IG hypothetical protein	2
OB2488 (166)	*Oceanobacillus ihenyensis* HTE831 (B)	Hypothetical conserved protein	1
ABC0230 (158)	*Bacillus clausii* KSM-K16 (B)	Unknown conserved protein	1
BH0678 (246)	*Bacillus halodurans* C-125 (B)	BH0678 protein	2
ABC4088 (142)	*Bacillus clausii* KSM-K16 (B)	Hypothetical protein	1
ExigDRAFT_1796 (161)	*Exiguobacterium sibiricum* 255-15 (B)	Hypothetical protein	1
OB3282 (155)	*Oceanobacillus ihenyensis* HTE831 (B)	Hypothetical conserved protein	1
BcerKBAB4DRAFT_2040 (241)	*Bacillus weihenstephanensis* KBAB4 (B)	Conserved hypothetical protein	2
B14911_09907 (144)	*Bacillus* sp. NRRL B-14911 (B)	Hypothetical protein	1
B14911_05359 (273)	*Bacillus* sp. NRRL B-14911 (B)	Hypothetical protein	2
BAA83944 (267)	*Bacillus halodurans* (B)	Unnamed protein product	2
BH1853 (158)	*Bacillus halodurans* C-125 (B)	Hypothetical protein	1
Bcer98DRAFT_3614 (177)	*Bacillus cereus* subsp. cytotoxis NVH 391-98 (B)	IG hypothetical protein	2
ExigDRAFT_0574 (253)	*Exiguobacterium sibiricum* 255-15 (B)	Hypothetical protein	2

**Table:** (g) List of proteins containing the 104-amino-acid-residue NTGFIG domain.

Gene ID (number of residues)	Organism	Description	Number of NTGFIG domains

BA2665 (232)	*Bacillus anthracis* str. *Ames* (B)	Hypothetical protein	2 tandem
BT9727_2444 (232)	*Bacillus thuringiensis* serovar konkukian str. 97-27 (B)	Hypothetical protein	2 tandem
BCZK2413 (232)	*Bacillus cereus* E33L (B)	Group-specific protein	2 tandem
BCE2700 (234)	*Bacillus cereus* ATCC 10987 (B)	Hypothetical protein	2 tandem
BC2674 (234)	*Bacillus cereus* ATCC 14579 (B)	Hypothetical protein	2 tandem
Bant_01003317 (236)	*Bacillus anthracis* str. A2012 (B)	Hypothetical protein	2 tandem
BCE_G9241_CNI_0263 (234)	*Bacillus cereus* G9241 (B)	Conserved hypothetical protein	2 tandem
BcerKBAB4DRAFT_0535 (232)	*Bacillus weihenstephanensis* KBAB4 (B)	Conserved hypothetical protein	2 tandem
Bcer98DRAFT_0128 (234)	*Bacillus cereus* subsp. cytotoxis NVH 391-98 (B)	Conserved hypothetical protein	2 tandem

**Table:** (h) List of proteins containing the 36-amino-acid-residue NxGK repeat.

Gene ID (number of residues)	Organism	Description and other known domains	Numbre of NxGK repeats

BA3686 (193)	*Bacillus anthracis* str. *Ames* (B)	Hypothetical protein, SAP domain (1)	2
BT9727_3378 (193)	*Bacillus thuringiensis* serovar konkukian str. 97-27 (B)	Hypothetical protein, SAP domain (1)	2
BCZK3328 (193)	*Bacillus cereus* E33L (B)	Hypothetical protein, SAP domain (1)	2
BC3626 (193)	*Bacillus cereus* ATCC 14579 (B)	Hypothetical protein, SAP domain (1)	2
BCE3645 (193)	*Bacillus cereus* ATCC 10987 (B)	Hypothetical protein, SAP domain (1)	2
RBTH_03615 (193)	*Bacillus thuringiensis* serovar israelensis ATCC 35646 (B)	Hypothetical cytosolic protein, SAP domain (1)	2
BCE_G9241_3579 (193)	*Bacillus cereus* G9241 (B)	Hypothetical cytosolic protein SAP domain (1)	2
BcerKBAB4DRAFT_0944 (193)	*Bacillus weihenstephanensis* KBAB4 (B)	Conserved hypothetical protein SAP domain (1)	2
B14911_25780 (189)	*Bacillus* sp. NRRL B-14911 (B)	Hypothetical protein SAP domain (1)	2

**Table:** (i) List of proteinscontaining the 95-amino-acid-residue VYV domain.

Gene ID (number of residues)	Organism	Description	Number of VYV domains

BA1701 (225)	*Bacillus anthracis* str. *Ames* (B)	Hypothetical protein	2 tandem
BAS1577 (227)	*Bacillus anthracis* str. Sterne (B)	Hypothetical protein	2 tandem
RBTH_03882 (1004)	*Bacillus thuringiensis* serovar israelensis ATCC 35646 (B)	Hypothetical exported protein	10 tandem
DSY3134 (1674)	*Desulfitobacterium hafniense Y51* (B)	Hypothetical protein	2 tandem

**Table:** (j) List of proteins containing the 75-amino-acid-residue KEWE domain.

Gene ID (number of residues)	Organism	Description	Number of KEWE domains

BA3147 (262)	*Bacillus anthracis* str. *Ames* (B)	Hypothetical protein	3 tandem
BAS2924 (344)	*Bacillus anthracis* str. Sterne (B)	Hypothetical protein	4 tandem
RBTH_06405 (331)	*Bacillus thuringiensis* serovar israelensis ATCC 35646 (B)	Hypothetical protein	4 tandem
pE33L466_0092 (328)	*Bacillus cereus* E33L (B)	Hypothetical protein	4 tandem
Bant_01003795 (178)	*Bacillus anthracis* str. A2012 (B)	Hypothetical protein	2 tandem
pBMB165 (247)	*Bacillus thuringiensis* serovar tenebrionis (B)	Hypothetical protein	3 tandem

**Table:** (k) List of proteins containing the 59-amino-acid-residue AFL domain.

Gene ID (number of residues)	Organism	Description	Number of AFL domains

BA3065 (290)	*Bacillus anthracis* str. *Ames* (B)	Hypothetical protein	2
BAS2851 (297)	*Bacillus anthracis* str. Sterne (B)	Hypothetical protein	2
Bant_01003715 (293)	*Bacillus anthracis* str. A2012 (B)	Hypothetical protein	2
RBTH_02124 (145)	*Bacillus thuringiensis* serovar israelensis ATCC 35646 (B)	Hypothetical protein	1
BcerKBAB4DRAFT_1832 (291)	*Bacillus weihenstephanensis* KBAB4 (B)	Conserved hypothetical protein	2

## Footnotes

^1^The multiple sequence alignments corresponding to representative repeats and domains from various proteins along with their GENE or SWall identifiers. (a) PxV domain, (b) FxF domain, (c) YEFF domain, (d) IMxxH domain, (e) VxxT domain, (f) ExW domain, (g) NTGFIG domain, (h) NxGK repeat (i) VYV domain, (j) KEWE domain, (k) AFL domain, (l) RIDVK repeat, (m)(a) AGQF repeat and (b) GSAL repeat. The numbers given in brackets indicate the start and end of amino-acid-residue positions corresponding to either the repeat or domain. The 80% consensus is labeled according to the alignment to the alignment generated at the website http://www.bork.embl-heidelberg.de/Alignment/consensus.html: alcohol (o, ST); aliphatic (I, ILV); any (., ACDEFGHIKLMNPQRSTVWY); aromatic (a, FHWY); charged (c, DEHKR); hydrophobic (h, ACFGHIKLMRTVWY); negative (−, DE); polar (p, CDEHKNQRST); positive (+, HKR); small (s, ACDGNPSTV); tiny (u, AGS); turn-like (t, ACDEGHKNQRST). A capital letter indicates 80% conservation of corresponding amino-acid residue. The secondary structure prediction indicated at the top was derived using the PHD program. Residues predicted with greater than 82% accuracy to form *β*-sheets are represented by “E” and *α*-helices are represented by “H.”
